# Racial and ethnic disparities in perceived health and barriers to care among adults with hip osteoarthritis: a national analysis of 16,000 + patients from the all of us program

**DOI:** 10.1007/s00402-026-06470-6

**Published:** 2026-08-24

**Authors:** Shujaa T. Khan, Sudhish A. Devadiga, Ahmed K. Emara, Smit Brahmbhatt, Khaled A Elmenawi, Peter G Delaney, Nicolas S. Piuzzi

**Affiliations:** Department of Orthopaedic Surgery, Cleveland Clinic, Cleveland, United States

**Keywords:** Health disparities, Access to care, Patient-provider communication, Social determinants of health

## Abstract

**Objective:**

To explore racial and ethnic disparities in perceived health, healthcare access, and perceived barriers among adults with hip osteoarthritis (OA) in a large, nationally representative U.S. cohort.

**Methods:**

Cross-sectional analysis of 16,743 adults with hip OA from the All of Us Research Program. Participants self-identified as Black, Hispanic, or White. Outcomes included self-reported general, mental, physical, and social health, provider communication, and structural barriers to care. Between-group comparisons used Chi-square tests.

**Results:**

Black and Hispanic participants were younger, had lower income and education, and relied more on Medicaid than White participants (all *p* < 0.001). They reported higher rates of unaffordable deductibles, difficulty accessing follow-up care, insurance coverage issues, transportation challenges, and medication cost-related nonadherence (all *p* < 0.001) Table 3. Minority groups also more often experienced poor provider respect, trust, and communication. Across all health domains, Black and Hispanic participants reported poorer outcomes and greater emotional distress (all *p* < 0.001).

**Conclusion:**

Significant racial and ethnic disparities exist in structural and perceptual barriers to care among adults with hip OA. These findings identify potentially modifiable barriers that warrant further study and may inform future efforts to improve equity in hip osteoarthritis care.

**Supplementary Information:**

The online version contains supplementary material available at 10.1007/s00402-026-06470-6.

## Introduction

 Hip osteoarthritis (OA) is a highly disabling condition affecting an estimated 7.2% of the global population and driving nearly 23.7 million ambulatory care visits annually [1, 2]. Projections suggest that, as obesity rates rise and populations age, hip OA prevalence and associated healthcare burden will continue to climb through at least 2030, further straining patients and the system alike [3]. Traditionally viewed as a biomechanical “wear-and-tear” disease, OA’s pathogenesis involves cartilage degradation, subchondral bone remodeling, synovial inflammation, and periarticular tissue changes within the joint as an organ [4, 5]. However, there is increasing recognition that social and structural determinants, such as socioeconomic status, insurance coverage, and geographic access, play key roles in who develops symptomatic disease, who obtains timely care, and who ultimately benefits from interventions [6–9].

Although overall incidence of hip OA is similar across racial and ethnic groups, Black and Hispanic adults bear a markedly greater burden of both structural joint damage and symptom severity. Meta-analysis shows that Black patients report pain scores nearly 0.6 standard deviations higher and disability scores 0.4 SD higher than non-Hispanic White patients, even after adjusting for age, sex, and body mass index [3]. Population cohorts such as Johnston County further demonstrate that Black participants have a higher prevalence of bilateral hip OA, more advanced radiographic features (including greater joint space narrowing and osteophyte formation), and faster progression to mobility impairment than their White peers, suggesting differences in disease severity and progression rather than disease onset [10]. These clinical gaps mirror, and are exacerbated by, disparities in care delivery. Black and Hispanic patients are less likely to receive diagnostic imaging, intra-articular injections, or total joint arthroplasty (TJA), even after controlling for income and insurance status [11]. National analyses report that African American and Hispanic patients have significantly lower odds of undergoing total hip arthroplasty (adjusted ORs ≈ 0.60–0.69) and total knee arthroplasty compared with White patients, a trend that has widened over the past decade [11]. In addition to these structural barriers such as high deductibles, transportation challenges, and limited specialist availability, perceptual barriers undermine minority patients’ engagement with care. Lower trust in providers, poorer communication, and concerns about discrimination or disrespect all contribute to delayed diagnosis and underuse of effective non-surgical treatments like NSAIDs, corticosteroid injections, and exercise programs [4].

Despite this robust literature, prior studies have largely evaluated structural barriers or patient experiences in isolation and often rely on adjusted models within more limited datasets. Few large, nationally representative cohorts have simultaneously examined both structural and perceptual dimensions of care in hip osteoarthritis. Understanding how these domains intersect is critical, as structural barriers and patient–provider interactions may jointly shape care utilization and outcomes. The All of Us Research Program, with its richly phenotyped, geographically diverse cohort and linkage of electronic health records to detailed patient surveys, offers a unique opportunity to fill this gap. Leveraging data from over 16,000 adults with hip OA, our study examines how social determinants (income, insurance, geography) and patient experiences (provider respect, communication, trust) converge to produce—and potentially perpetuate—racial and ethnic disparities in osteoarthritis management. By identifying modifiable barriers and perceptual factors, we aim to inform culturally sensitive, system-level interventions that promote equitable outcomes in hip OA care.

To organize the present analysis, we developed a conceptual framework in which racial and ethnic differences in self-reported health may be related to sociodemographic characteristics, structural barriers to care, and patient–provider interactions. These factors may separately or jointly relate to perceived health and symptom burden. The framework guided the organization of the variables examined in this study. These proposed relationships were not evaluated as causal or mediating pathways. The purpose of this exploratory study was to examine racial and ethnic differences in perceived health, healthcare access, patient–provider interactions, and structural barriers to care among adults with hip osteoarthritis in the All of Us Research Program.

## Methods

### Study design and setting

We conducted a cross-sectional analysis using data from the All of Us Research Program (v8 Controlled Tier, data release February 2024), a large, diverse, nationwide cohort designed to accelerate precision medicine. All of Us enrolls participants from health care provider organizations, community health centers, and direct volunteers across the United States. Data includes electronic health records, survey responses, and physical measurements. The All of Us Research Program obtained informed consent from all participants and received institutional review board approval at participating sites.

### Study population

Participants were eligible if they were ≥ 18 years old and had at least one diagnosis code for hip osteoarthritis (International Classification of Diseases, Tenth Revision [ICD-10] M16.x) in their electronic health record and complete self-reported demographic and survey data for the variables of interest. We excluded participants with missing race/ethnicity or incomplete survey responses on provider communication, barriers to care, or health status measures. The final analytic sample comprised 16,743 individuals, stratified by self-identified race/ethnicity as Black (*n* = 3,545), Hispanic (*n* = 1,637), or White (*n* = 11,651). The participant selection process is shown in the flow diagram (Supplementary Fig. 1). Because completion varied across the All of Us surveys, the number of participants included differed by outcome domain. The sample size for each domain is reported in the corresponding Results subsection and table.

### Variables

The primary exposure was self-reported race/ethnicity (Black, Hispanic, White). We also captured age group (18–44, 45–64, ≥ 65 years), sex at birth, body mass index (BMI) category (underweight to obesity class III), educational attainment (< high school to advanced degree), employment status (employed, unemployed, disabled, retired, other), household income (<$25 000 to ≥$100 000), marital status (married, partner, never married, divorced, separated, widowed), and insurance type (private, Medicare, Medicaid, other).

### Outcomes of interest

We evaluated three domains. Provider communication and respect included ease of understanding advice, feeling respected, and experiencing disrespect (e.g., being talked down to or not listened to). Structural barriers covered high deductibles, follow-up access, insurance acceptance by providers, out-of-pocket costs, transportation issues, and medication nonadherence due to cost. Response options for provider communication and barriers items were categorical (e.g., Never, Rarely, Sometimes, Most of the Time, Always; Yes/No). Health status encompassed self-rated general, mental, physical, and social health (Excellent-Poor), fatigue (5-point scale) and pain severity (0–10 scale) over seven days, and frequency of emotional problems (Never-Always).

### Statistical analysis

All analyses were performed in R version 4.3. Descriptive statistics (frequencies and percentages) summarized participant characteristics and outcome measures by race/ethnicity. Between-group comparisons used Pearson’s chi-square tests for categorical variables. A two-sided *p* < 0.05 was considered statistically significant. No adjustments for multiple comparisons were made given the exploratory, hypothesis-generating nature of this analysis.

Given the exploratory and descriptive aims of this study, analyses were not adjusted for covariates. However, differences in sociodemographic characteristics across racial and ethnic groups were considered in interpretation, and future analyses will evaluate adjusted associations.

This study is reported in accordance with the STROBE guidelines (see Supplementary Table 1).

## Results

### Study population and demographic characteristics

Significant differences were observed across all measured sociodemographic variables (all *p* < 0.001) (Table 1). Black and Hispanic patients were younger: 47.7 and 44.8% were aged 18–64 years versus 25.3% of White patients. Female sex predominated in minority groups (69.1% Black; 72.3% Hispanic) compared with 62.3% of White patients. Black and Hispanic patients also had lower educational attainment (13.4% of Black and 32.6% of Hispanic patients had less than a high school diploma versus 2.0% of White patients) and were more likely to report annual incomes below $25,000 (62.4% Black; 58.7% Hispanic; 16.6% White). Employment status differed markedly: 33.5% of Black and 29.2% of Hispanic patients were disabled compared with 11.2% of White patients, while retirement was more common among White participants (50.1%). Insurance coverage patterns further highlighted disparities: Medicaid covered 32.8% of Black and 31.5% of Hispanic patients but only 5.7% of White patients, whereas private insurance was held by 44.4% of White versus 18.7% of Black and 18.3% of Hispanic patients.

**Table 1:** Demographic and socioeconomic characteristics of adults with hip osteoarthritis, by race and ethnicity

Category	Black (*n* = 3545)	Hispanic (*n* = 1637)	White (*n* = 11651)	*P*-value
BMI category				< 0.001
Underweight	1.6% (57)	1.2% (20)	1.3% (151)	
Normal weight	15.5% (549)	17.7% (290)	24.9% (2901)	
Overweight	24.9% (883)	32.7% (535)	32.3% (3763)	
Obesity I	23.0% (815)	26.9% (440)	22.1% (2575)	
Obesity II	16.9% (599)	13.1% (214)	10.9% (1270)	
Obesity III	18.0% (638)	8.4% (138)	8.5% (990)	
Age group				< 0.001
18–44 years	4.0% (142)	5.8% (95)	2.7% (315)	
45–64 years	43.7% (1549)	39.0% (638)	22.6% (2633)	
65 + years	52.3% (1854)	55.2% (904)	74.7% (8703)	
Sex at birth				
Female	69.1% (2450)	72.3% (1184)	62.3% (7259)	
Male	30.9% (1095)	27.7% (453)	37.7% (4392)	
Education				< 0.001
< High school	13.4% (475)	32.6% (534)	2.0% (233)	
High school or GED	30.3% (1074)	22.5% (368)	12.2% (1421)	
Some college	34.5% (1223)	24.0% (393)	27.9% (3251)	
College graduate	13.2% (468)	14.3% (234)	26.5% (3088)	
Advanced degree	8.5% (301)	6.6% (108)	31.4% (3658)	
Employment status				< 0.001
Employed	24.3% (861)	24.5% (401)	30.7% (3577)	
Unemployed	9.8% (347)	8.7% (142)	4.5% (524)	
Disabled	33.5% (1188)	29.2% (478)	11.2% (1305)	
Retired	29.0% (1028)	30.5% (499)	50.1% (5837)	
Other	3.3% (117)	7.1% (116)	3.4% (396)	
Income				< 0.001
<$25k	62.4% (2212)	58.7% (961)	16.6% (1934)	
$25k–49k	17.9% (635)	17.4% (285)	19.2% (2237)	
$50k–74k	9.2% (326)	10.3% (169)	16.6% (1934)	
$75k–99k	4.6% (163)	4.5% (74)	14.3% (1666)	
≥$100k	5.9% (209)	9.2% (151)	33.4% (3891)	
Marital status				< 0.001
Married	24.4% (865)	38.5% (630)	56.8% (6618)	
Living with partner	3.6% (128)	3.8% (62)	2.9% (338)	
Never married	30.1% (1067)	15.1% (247)	10.3% (1200)	
Divorced	23.6% (837)	23.5% (385)	17.6% (2051)	
Separated	6.7% (238)	8.0% (131)	1.4% (163)	
Widowed	11.6% (411)	11.1% (182)	9.9% (1153)	
Insurance type				< 0.001
Private insurance	18.7% (663)	18.3% (300)	44.4% (5173)	
Medicare	40.2% (1425)	37.0% (606)	44.0% (5126)	
Medicaid	32.8% (1163)	31.5% (516)	7.5% (874)	
Other	8.3% (294)	13.2% (216)	5.9% (687)	

*BMI* body mass index

*p values calculated using Pearson’s chi-square test

### Patient–provider communication and respect

Among those who responded to care‑experience items (n=9,616), Black and Hispanic participants reported more challenges in understanding and feeling respected by providers (Table 2). Although a majority across all groups “always” found health advice easy to understand, this was highest among Black patients (69.5%) and lowest among White patients (62.9%) (*p*<0.001). However, Black patients more frequently reported being treated with less respect: 20.0% “sometimes” or more often felt treated disrespectfully versus 7.8% of White and 14.1% of Hispanic patients (*p*<0.001). Similarly, 9.5% of Black patients “sometimes” or more often felt that their doctor did not listen, compared with 3.6% of White patients (*p*<0.001).

**Table 2.** Patient–provider communication and perceived respect among adults with hip osteoarthritis, stratified by race and ethnicity

Group	Category	Black (n=830)	Hispanic (n=368)	White (n=7418)	p_value
Health advice: ease of understanding	Always	69.5% (577)	66.9% (246)	62.9% (4666)	<0.001
	Most of the time	29.6% (246)	30.5% (112)	36.7% (2722)	
	None of the time	0.9% (7)	2.6% (10)	0.4% (30)	
Health advice: respected by provider	Always	82.8% (687)	84.6% (311)	80.6% (5979)	0.114
	Most of the time	16.9% (140)	15.0% (55)	19.3% (1432)	
	None of the time	0.4% (3)	0.4% (1)	0.2% (15)	
How often treated with less respect	Never	44.8% (372)	61.3% (226)	61.4% (4555)	<0.001
	Rarely	31.7% (263)	22.4% (82)	29.1% (2159)	
	Sometimes	20.0% (166)	14.1% (52)	7.8% (579)	
	Most of the time	2.4% (20)	1.9% (7)	0.5% (37)	
	Always	1.1% (9)	0.3% (1)	1.1% (82)	
How often doctor acts better than you	Never	63.5% (527)	72.3% (266)	59.6% (4421)	<0.001
	Rarely	20.2% (168)	15.8% (58)	27.6% (2047)	
	Sometimes	12.4% (103)	9.2% (34)	11.1% (823)	
	Most of the time	2.9% (24)	1.9% (7)	1.2% (89)	
	Always	1.0% (8)	0.8% (3)	0.6% (45)	
How often doctor does not listen	Never	43.3% (359)	55.0% (202)	39.2% (2908)	<0.001
	Rarely	25.0% (208)	18.6% (68)	34.6% (2567)	
	Sometimes	23.4% (194)	20.0% (74)	22.6% (1676)	
	Most of the time	6.0% (50)	5.3% (20)	2.7% (200)	
	Always	2.3% (19)	1.1% (4)	0.9% (67)	

*p values calculated using Pearson’s chi-square test

### Structural barriers to care

Data on barriers (n=8,569) revealed that Black and Hispanic patients faced significantly greater structural impediments than White patients (Table 3). High deductibles were reported by 10.7% of Black and 9.1% of Hispanic patients versus 6.1% of White patients (*p*<0.001). Difficulties accessing follow‑up care affected 10.9% of Black and 10.4% of Hispanic versus 5.1% of White patients (*p*<0.001). Black patients were twice as likely to skip medications to save money (10.9% vs. 5.7% of White patients; *p*<0.001) and almost three times as likely to report transportation issues (17.1% vs. 5.6%; *p*<0.001). Need to see a specialist but facing access issues was reported by 13.2% of Black and 10.8% of Hispanic versus 6.9% of White patients (*p*<0.001).

**Table 3:** Structural barriers to healthcare access reported by adults with hip osteoarthritis, by race and ethnicity

Variable	Barrier present?	Black (*n* = 1,062)	Hispanic (*n* = 509)	White (*n* = 6,998)	*p*-value
	No	89.3% (948)	90.9% (463)	93.9% (6571)	< 0.001
Deductible too high	Yes	10.7% (114)	9.1% (46)	6.1% (427)	< 0.001
Difficulty getting follow-up care	Yes	10.9% (116)	10.4% (53)	5.1% (357)	< 0.001
	No	89.1% (946)	89.6% (456)	94.9% (6641)	< 0.001
Had to pay out of pocket	Yes	14.9% (158)	13.6% (69)	12.4% (868)	0.078
	No	85.1% (904)	86.4% (440)	87.6% (6130)	0.078
Difficulty finding a healthcare provider	Yes	9.2% (98)	7.4% (38)	3.6% (252)	< 0.001
	No	90.8% (964)	92.6% (471)	96.4% (6746)	< 0.001
Insurance not accepted	Yes	12.6% (134)	12.3% (63)	8.4% (588)	< 0.001
	No	87.4% (928)	87.7% (446)	91.6% (6410)	< 0.001
Nervous about getting care	Yes	9.7% (103)	10.1% (51)	8.0% (560)	0.042
	No	90.3% (959)	89.9% (458)	92.0% (6438)	0.042
Trouble paying for prescription medicines	Yes	20.9% (222)	17.8% (91)	9.3% (651)	< 0.001
	No	79.1% (840)	82.2% (418)	90.7% (6347)	< 0.001
Rural area	Yes	5.4% (57)	5.1% (26)	2.5% (175)	< 0.001
	No	94.6% (1005)	95.2% (485)	97.5% (6823)	< 0.001
Skipped medication to save money	Yes	10.9% (116)	9.2% (47)	5.7% (399)	< 0.001
	No	89.1% (946)	90.8% (462)	94.3% (6599)	< 0.001
Trouble seeing a specialist	Yes	13.2% (140)	10.8% (55)	6.9% (483)	< 0.001
	No	86.8% (922)	89.2% (454)	93.1% (6515)	< 0.001
Could not take time off work	Yes	6.5% (69)	9.0% (46)	4.7% (329)	< 0.001
	No	93.5% (993)	91.0% (463)	95.3% (6669)	< 0.001
Took less medication to save money	Yes	11.2% (119)	8.8% (45)	6.5% (455)	< 0.001
	No	88.8% (943)	91.2% (464)	93.5% (6543)	< 0.001
Transportation problems	Yes	17.1% (182)	15.7% (80)	5.6% (392)	< 0.001
	No	82.9% (880)	84.3% (429)	94.4% (6606)	< 0.001

*p values calculated using Pearson’s chi-square test

### Health status and symptom burden

Assessment of general, mental, physical, and social health (n=9,078) demonstrated significantly poorer outcomes among Black and Hispanic patients across nearly all domains (Table 4). In the prior 7 days, moderate to severe fatigue was more common among Black (41.9%) and Hispanic (45.8%) than White patients (38.2%) (*p*<0.001), and severe to very severe pain (scores ≥7) affected 25.6% of Black, 25.0% of Hispanic, and only 13.9% of White patients (*p*<0.001). Emotional distress (“often” or“always”) was reported by 18.6% of Black and 20.9% of Hispanic participants versus 11.6% of White participants (*p*<0.001). In self‑rated general health, only 21.1% of Black and 19.1% of Hispanic patients described their health as “very good” or “excellent,” compared with 41.2% of White patients (*p*<0.001). Similar patterns were observed in mental, physical, and social health ratings, with minority groups more frequently endorsing “fair” or “poor” status across these dimensions (all *p*<0.001).

**Table 4:** Self-reported health status, symptom burden, and emotional distress among adults with hip osteoarthritis, stratified by race and ethnicity

Question	Answer	Black (%) (*n* = 1,068)	Hispanic (%) (*n* = 512)	White (%) (*n* = 7,498)	*p*-value
Average fatigue 7 days	None	16.4% (175)	17.4% (89)	17.9% (1342)	< 0.001
	Mild	32.3% (345)	26.8% (137)	42.8% (3209)	< 0.001
	Moderate	38.5% (411)	37.7% (193)	30.1% (2257)	< 0.001
	Severe	9.5% (101)	13.1% (67)	7.5% (562)	< 0.001
	Very Severe	3.4% (36)	5.1% (26)	1.7% (127)	< 0.001
Average pain 7 days	0	7.8% (83)	10.6% (54)	11.8% (885)	< 0.001
	1	4.5% (48)	4.9% (25)	15.8% (1185)	< 0.001
	2	5.6% (60)	5.7% (29)	13.6% (1020)	< 0.001
	3	7.3% (78)	7.0% (36)	11.7% (877)	< 0.001
	4	8.2% (88)	6.6% (34)	7.8% (585)	< 0.001
	5	10.7% (114)	10.5% (54)	8.3% (622)	< 0.001
	6	9.6% (103)	9.0% (46)	7.3% (547)	< 0.001
	7	12.5% (134)	11.4% (58)	6.6% (495)	< 0.001
	8	13.1% (140)	13.3% (68)	4.8% (360)	< 0.001
	9	5.2% (56)	5.1% (26)	1.8% (135)	< 0.001
	10	7.5% (80)	7.2% (37)	1.6% (120)	< 0.001
Emotional problem 7 days	Never	25.2% (269)	25.6% (131)	25.5% (1912)	< 0.001
	Rarely	22.6% (241)	20.5% (105)	32.7% (2452)	< 0.001
	Sometimes	33.6% (359)	33.0% (169)	28.6% (2144)	< 0.001
	Often	12.1% (129)	14.1% (72)	9.2% (690)	< 0.001
	Always	6.5% (69)	6.8% (35)	2.4% (180)	< 0.001
General health	Excellent	4.2% (45)	4.8% (25)	8.5% (637)	< 0.001
	Very Good	16.9% (180)	14.2% (73)	32.7% (2452)	< 0.001
	Good	37.6% (402)	34.1% (175)	36.3% (2722)	< 0.001
	Fair	34.0% (363)	36.0% (184)	17.9% (1342)	< 0.001
	Poor	7.2% (77)	10.9% (56)	4.7% (352)	< 0.001
General mental health	Excellent	12.4% (132)	11.5% (59)	18.2% (1365)	< 0.001
	Very Good	26.5% (283)	22.7% (116)	36.9% (2767)	< 0.001
	Good	30.9% (330)	34.4% (176)	26.2% (1964)	< 0.001
	Fair	13.9% (148)	21.9% (112)	10.1% (757)	< 0.001
	Poor	4.0% (43)	5.5% (28)	2.0% (150)	< 0.001
General physical health	Excellent	3.8% (41)	4.4% (23)	7.1% (532)	< 0.001
	Very Good	14.7% (157)	12.9% (66)	29.8% (2234)	< 0.001
	Good	16.9% (180)	16.7% (86)	27.9% (2092)	< 0.001
	Fair	26.7% (285)	24.2% (124)	37.2% (2789)	< 0.001
	Poor	32.7% (349)	32.8% (168)	23.4% (1755)	< 0.001
General social	Excellent	18.0% (192)	19.1% (98)	8.8% (660)	< 0.001
	Very Good	5.7% (61)	7.2% (37)	2.7% (202)	< 0.001
	Good	14.0% (150)	17.5% (90)	20.7% (1552)	< 0.001
	Fair	25.4% (271)	24.8% (127)	36.4% (2729)	< 0.001
	Poor	34.1% (364)	36.1% (185)	25.5% (1912)	< 0.001
Social satisfaction	Excellent	20.5% (219)	16.4% (84)	12.3% (922)	< 0.001
	Very Good	6.0% (64)	5.3% (27)	5.1% (382)	< 0.001
	Good	16.4% (175)	17.4% (89)	17.9% (1342)	< 0.001
	Fair	32.3% (345)	26.8% (137)	42.8% (3209)	< 0.001
	Poor	38.5% (411)	37.7% (193)	30.1% (2257)	< 0.001

*p values calculated using Pearson’s chi-square test

## Discussion

Racial and ethnic disparities in osteoarthritis (OA) care are well-documented, but few studies have examined the full range of structural and perceptual barriers faced by diverse populations with hip OA across the United States. In this national cohort of 16,743 adults with hip OA, we found that Black and Hispanic participants reported poorer general, mental, physical, and social health and faced substantially more structural (cost, insurance acceptance, transportation, specialist access) and perceptual (respect, listening, communication) barriers than White participants. These factors are likely related rather than independent. Financial and transportation barriers, for example, may limit access to treatment, while negative interactions with clinicians may affect trust and engagement with care. However, the cross-sectional and unadjusted design does not allow us to determine the relative contribution of these factors. Adjusted and longitudinal studies are needed to examine which factors are most closely associated with symptoms, healthcare use, and treatment outcomes.

Transportation difficulties are one component of the gap: the rate among White patients in our sample (5.6%) was similar to that reported for a predominantly White orthopedic clinic by Bernstein et al., but it was nearly three times higher among Black patients (17.1%) [12]. Cost-related barriers showed a similar pattern. Trouble paying for medications affected 9.3% of White patients in our cohort, more than double the ~4% observed by Bernstein et al., and was markedly higher among Black patients (20.9%) [12]. Similar to Hausmann et al., who showed that perceived racism was linked to lower ratings of provider warmth/respectfulness and communication ease among OA patients, our data show Black patients were more than twice as likely as White patients to feel disrespected or unheard [13]. Structural barriers are compounded by communication gaps; prior work found that minority patients with arthritis reported feeling less listened to and less involved in shared decision making, suggesting provider-patient dynamics also play a role in perpetuating disparities [14]. Moreover, communication barriers tied to language and education described by Diaz et al. align with our finding that minority groups, who also had lower educational attainment, more often reported difficulty understanding or trusting providers [15]. Beyond perception, our results echo system-level inequities: like Yi et al [16] and Amen et al.[17], who documented lower arthroplasty receipt and worsening perioperative disparities in socially vulnerable and Black communities, we observed upstream obstacles (insurance denials, high deductibles, follow-up access) that plausibly contribute to those downstream surgical gaps. Psychosocial pathways highlighted by Jin & Yarns [18] and Derricks et al. [19] (anger, perceived injustice, and discrimination) map onto the elevated emotional distress and pain severity we observed, suggesting that stress-mediated amplification of symptoms may be an underrecognized driver of racial differences in hip OA burden. Psychosocial variables such as affect, arthritis self-efficacy, and emotion-focused coping strategies may partly explain racial disparities in OA-related pain and function and are potentially modifiable through behavioral interventions [14].

Clinically, these findings suggest that routine screening for cost, transportation, and medication access during hip OA visits, with referral to navigators or social workers when appropriate, may be worth further study. This may be especially relevant given evidence that these disparities can persist over time; a longitudinal study of knee OA found that Black participants reported greater pain and disability than White participants over nine years, even after adjustment for socioeconomic and clinical factors [20]. Communication training and efforts to diversify the workforce may also help improve trust and understanding. Other potential approaches include telehealth and ride-share support, streamlined prior authorization, and incorporating equity-focused communication and barrier screening into value-based reimbursement. Policy options that warrant further evaluation include Medicaid reforms, such as lower deductibles and broader specialist networks, and investment in community-based rehabilitation programs in highly vulnerable areas. However, because the present study was exploratory and unadjusted, these findings do not establish the effectiveness of any specific intervention. Future work should quantify the relative contribution of structural and perceptual barriers through adjusted, mediation, or path analyses; follow patients longitudinally to determine whether early barriers are associated with delayed arthroplasty and long-term outcomes; and test pragmatic interventions such as transport vouchers, cost offsets, and communication coaching. Together, these steps may help translate the disparities identified in this study into more targeted strategies to improve equity in hip OA care.

The findings of this study should be viewed in the context of its limitations. First, its cross-sectional design precludes causal inferences between race/ethnicity, barriers to care, and health outcomes. Additionally, we did not perform adjusted analyses to account for differences in sociodemographic characteristics across groups. As such, observed associations may be partially confounded by these factors. Second, all measures were self-reported and subject to recall or reporting bias, which may vary across cultural or educational backgrounds. Third, hip OA was identified using ICD-10 codes without confirmation of disease severity, laterality, or treatment history, which may influence symptom burden and care experiences. Fourth, complete-case analysis may introduce selection bias if excluded participants differed systematically from those included. Finally, while the All of Us cohort is diverse, it is not a probability sample of the U.S. population, and survey items were not specific to orthopedic care, which may limit generalizability and content specificity. These limitations should temper interpretation but do not diminish the clear pattern of multifaceted disparities observed.

## Conclusion

Racial and ethnic minority adults with hip osteoarthritis experience a heavier symptom burden and encounter more obstacles to getting care. These obstacles are not isolated shortcomings but interconnected features of how care is financed, delivered, and communicated. These findings identify structural and interpersonal factors that warrant further study as potential targets for improving equitable access to hip osteoarthritis care.

## Supplementary Information

Below is the link to the electronic supplementary material.

Supplementary Material 1

## Data Availability

No datasets were generated or analysed during the current study.
